# Proximity‐Induced Ferroelectric Switching in Wurtzite/Fluorite Bilayers for High‐Performance Ferroelectric Field‐Effect Transistor

**DOI:** 10.1002/adma.202509088

**Published:** 2025-08-21

**Authors:** Kyung Do Kim, Min Kyu Yeom, Han Sol Park, Gwangsik Jeon, Cheol Seong Hwang

**Affiliations:** ^1^ Department of Materials Science and Engineering and Inter‐University Semiconductor Research Center Seoul National University Seoul 08826 South Korea

**Keywords:** aluminum scandium nitride, ferroelectric, ferroelectric field‐effect transistors, hafnium zirconium oxide, heterostructure, proximity ferroelectricity

## Abstract

Proximity ferroelectricity, wherein polarization switching in one ferroelectric layer with a lower energy barrier can trigger switching in an adjacent ferroelectric with a higher energy barrier, has been demonstrated only in bilayers composed of structurally similar wurtzite‐structured materials. This work demonstrates proximity‐induced ferroelectric switching across a heterostructure composed of crystallographically and functionally dissimilar materials, wurtzite‐structured AlScN and fluorite‐structured HfZrO_2_. The AlScN/HfZrO_2_ and AlN/HfZrO_2_ bilayers exhibit cooperative switching dynamics despite their contrasting symmetry and polarization behaviors. This interfacial coupling produces a unique ferroelectric response, characterized by low remanent polarization and a high coercive field. Using the AlN/HfZrO_2_ bilayer, a ferroelectric field‐effect transistor is fabricated on a p‐type Si substrate with phosphorus‐doped source and drain regions. The device shows a wide memory window with excellent retention and endurance. These results establish a versatile materials design strategy for engineering ferroelectricity via structural and functional heterogeneity.

## Graphical abstract

Proximity‐induced switching in AlScN/HfZrO_2_ and AlN/HfZrO_2_ bilayers enables a novel combination of low remanent polarization and high coercive field. A ferroelectric field‐effect transistor incorporating an AlN/HfZrO_2_ gate stack yields a wide memory window, superior retention, and endurance characteristics. This work pioneers a new strategy for engineering ferroelectric response through integrating distinct ferroelectric materials.

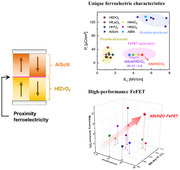

## 1 Introduction

Ferroelectric materials, characterized by their switchable spontaneous polarization, are fundamental in various electronic applications, including non‐volatile memories, sensors, and actuators.^[^ ^1^ ^]^ Among these, ferroelectric field‐effect transistors (FeFETs) are promising candidates for next‐generation non‐volatile memory.^[^ ^2^ ^]^ Their memory window and long‐term stability are primarily governed by two intrinsic properties of the ferroelectric layer: the remanent polarization (*P*_r_) and the coercive field (*E*_C_). Therefore, achieving an optimal combination of these parameters is crucial to ensure robust and efficient device operation.^[^ ^3^ ^]^ Fluorite‐structured ferroelectrics, such as hafnium zirconium oxide (HfZrO_2_), have become mainstream due to their scalability and compatibility with complementary metal‐oxide‐semiconductor (CMOS) technology.^[^ ^4^ ^]^ These materials typically exhibit a *P*_r_ of 20–30 µC cm^−^^2^ and an *E*_C_ of ≈1 MV cm^−1^, enabling low‐voltage operation but inherently limiting the FeFET memory window, which scales with the double coercive voltage (2V_C_).^[^ ^3a^ ^]^ In contrast, wurtzite‐structured ferroelectrics, such as aluminum scandium nitride (AlScN), offer high *E*_C_ (>6 MV cm^−1^), theoretically enabling expanded memory windows.^[^ ^5^ ^]^ Jariwala et al. demonstrated the feasibility of AlScN material in FeFET devices by utilizing 2D channels.^[^ ^6^ ^]^ Nevertheless, their ultrahigh *P*_r_ (>100 µC cm^−^^2^) can generate strong depolarization fields, promoting charge injection and interfacial trapping that degrade memory stability, and their applicability to Si channel has not been reported. So far, no ferroelectric material has achieved the ideal ferroelectric properties required for FeFETs.

A novel approach to overcoming this limitation involves heterostructure design via “proximity ferroelectricity,” wherein switching in one ferroelectric layer induces ferroelectric switching in an adjacent layer through strong interfacial coupling.^[^ ^7^ ^]^ Electric and elastic fields at domain walls can propagate across the interface, locally lowering the switching barrier in the neighboring layer. While such proximity‐induced switching has been demonstrated in wurtzite bilayers, its applicability across structurally dissimilar ferroelectrics remains unclear.

This study demonstrates proximity‐induced switching in an AlScN/HfZrO_2_ bilayer. Despite structural dissimilarity, polarization switching initiated in the HfZrO_2_ film induced polarization reversal in the AlScN film, confirming the presence of interfacial proximity effects. This cooperative switching enabled the bilayer to exhibit low *P*_r_ and high *E*_C_. Notably, this mechanism also facilitated switching in undoped AlN, which is typically non‐switchable due to its high switching barrier.^[^ ^8^ ^]^ To evaluate the applicability of such bilayers in FeFET applications, devices were fabricated using a gate‐last process with a p‐type Si channel, where the source and drain regions were doped with phosphorus. When implemented as a FeFET gate stack, the AlN/HfZrO_2_ bilayer yielded a wide memory window of 10.9 V with excellent stability. These findings establish a new paradigm for engineering ferroelectric response by integrating distinct ferroelectric materials.

## 2 Ferroelectric Switching Characteristics of Al_2_O_3_/HfZrO_2_ and AlScN/HfZrO_2_ Bilayers

**Figure 1:**
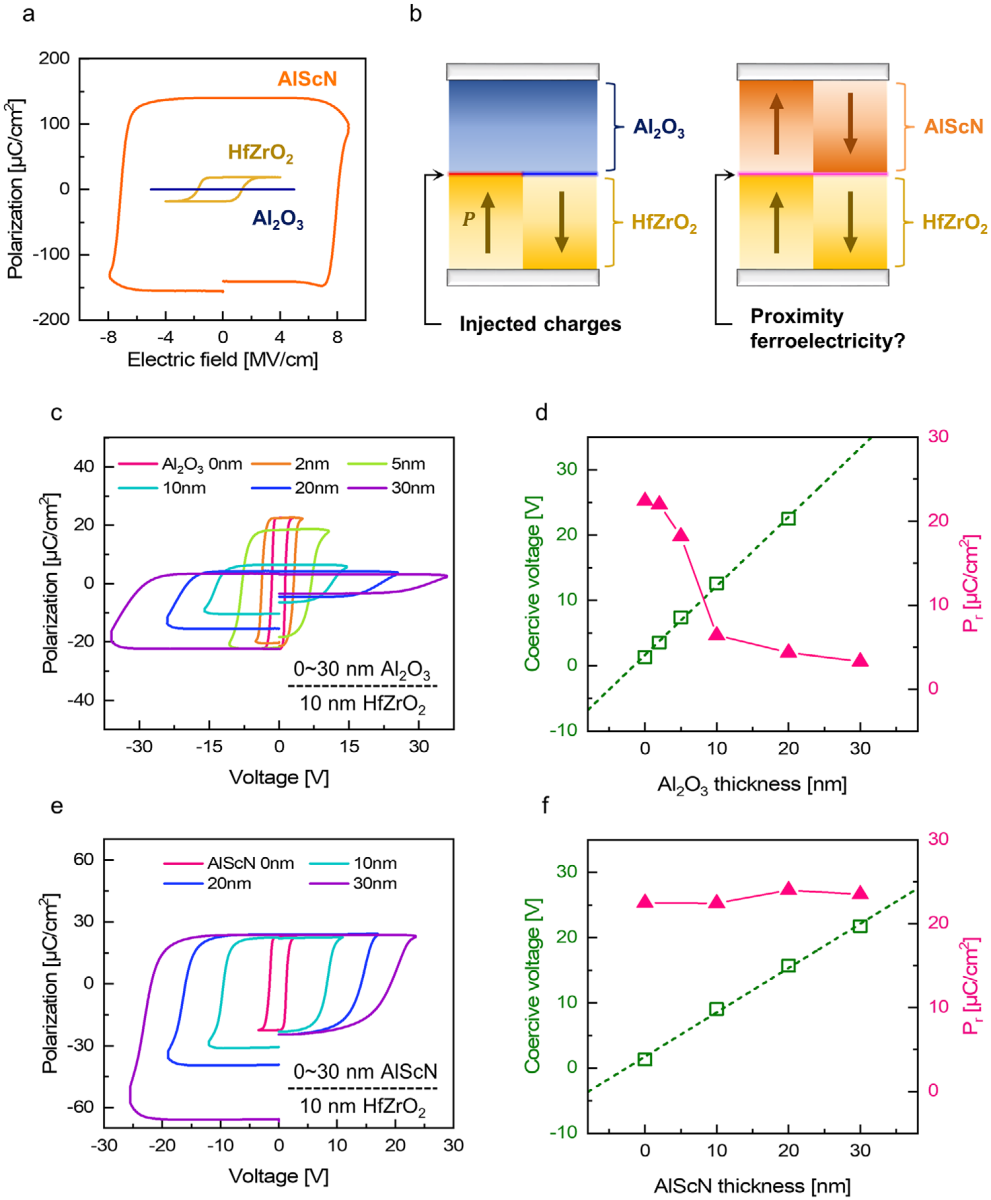
a) *P*–*E* curves of HfZrO_2_, Al_2_O_3_, and AlScN films measured using the triangular PUND method. Clear ferroelectric switching was observed in HfZrO_2_ and AlScN films, while the Al_2_O_3_ film exhibited no polarization hysteresis. b) Schematic illustration of ferroelectric switching mechanism in Al_2_O_3_/HfZrO_2_ and AlScN/HfZrO_2_ bilayers, highlighting the distinct charge screening behaviors at the interfaces. c) *P–V* curves of the Al_2_O_3_/HfZrO_2_ bilayer and d) dependence of *V*_C_ and *P*_r_ on Al_2_O_3_ film thickness. e) *P–V* curves of the AlScN/HfZrO_2_ bilayer and f) dependence of *V*_C_ and *P*r on AlScN film thickness.

**Figure** **1**a shows the polarization‐electric field (P–E) hysteresis curves of 10 nm thick HfZrO_2_, 30 nm thick Al_2_O_3_, and 30 nm thick AlScN films, measured using the positive‐up‐negative‐down (PUND) method with 50 kHz triangular pulses. The AlScN film was deposited with *x* = 0.2 in Al_1‐_ *_x_* Sc*_x_* N, and the HfZrO_2_ film was prepared with a 1:1 Hf:Zr ratio. The Experimental section provides deposition details.

The Al_2_O_3_ film exhibited typical dielectric behavior without hysteresis, whereas the HfZrO_2_ and AlScN films showed clear ferroelectric switching. AlScN exhibited significantly higher *P*_r_ and *E*_C_ than HfZrO_2_ and a steeper switching near *E*_C_, highlighting their distinct ferroelectric properties.^[^ ^9^ ^]^

Before evaluating the AlScN (or AlN)/HfZrO_2_ bilayer, the switching behavior of HfZrO_2_ in contact with a dielectric Al_2_O_3_ layer was investigated. In the metal‐dielectric‐ferroelectric‐metal (MDFM) structure, ferroelectric charges are compensated by injected charges through the dielectric layer, as illustrated in Figure 1b.^[^ ^10^ ^]^ To validate this mechanism, TiN/Al_2_O_3_/HfZrO_2_/TiN capacitors were fabricated, with a HfZrO_2_ thickness of 10 nm and an Al_2_O_3_ thickness varying from 0 to 30 nm. Grazing‐incidence X‐ray diffraction (GIXRD) patterns confirmed minimal effect of the Al_2_O_3_ film on the crystallization of the HfZrO_2_ film, as shown in Figure S1a of the Supporting Information.

Figure 1c shows the polarization–voltage (*P*–*V*) hysteresis loops of Al_2_O_3_/HfZrO_2_ bilayers with varying Al_2_O_3_ thicknesses. Due to the influence of leakage current under negative bias, *P*_r_ values were extracted from the positive bias region of the *P*–*V* curves. *V*_C_ was determined from the switching peak in the current density–voltage (*J*–*V*) curve (Figure S1b of the Supporting Information).

Figure 1d summarizes the *P*_r_ and *V*_C_ evolution as a function of Al_2_O_3_ thickness. *V*_C_ increased linearly with increasing Al_2_O_3_ thickness due to the charge injection through the Al_2_O_3_ film during HfZrO_2_ ferroelectric switching.^[^ ^10b^ ^]^ However, the amount of injected charge decreased with increasing Al_2_O_3_ thickness, resulting in limited switching and a corresponding reduction in *P*_r_.^[^ ^10^, ^11^ ^]^ As a result, the *P*_r_ decreased significantly from ≈22 µC cm^−2^ with the single HfZrO_2_ layer to 3 µC cm^−2^ at an Al_2_O_3_ thickness of 30 nm. These results indicate that in the MDFM structure, the dielectric layer's charge‐storing capacity is insufficient to screen the ferroelectric polarization charges, making charge injection play a dominant role in modulating the switching characteristics.

Then, TiN/AlScN/HfZrO_2_/TiN capacitors were fabricated, maintaining a HfZrO_2_ thickness of 10 nm while varying the AlScN film thickness from 0 to 30 nm. GIXRD patterns confirmed that changes in AlScN thickness had a negligible impact on the HfZrO_2_ film crystallization (Figure S1c of the Supporting Information). Further structural characterization using high‐resolution transmission electron microscopy (HRTEM) and energy‐dispersive X‐ray spectroscopy (EDS) mapping revealed that AlScN and HfZrO_2_ films were well‐crystallized, exhibiting sharp interfaces without interdiffusion (Figure S2 of the Supporting Information).

Figure 1e and Figure S1d of the Supporting Information show the evolution of *P*–*V* and *J–V* curves of the AlScN/HfZrO_2_ bilayers with varying AlScN thickness. *V*_C_ increased linearly with AlScN thickness, whereas *P*_r_ remained constant with the value of the HfZrO_2_ single film (≈22 µC cm^−2^), as shown in Figure 1f. This behavior contrasts with the Al_2_O_3_/HfZrO_2_ system, suggesting a different polarization switching mechanism.

Proximity ferroelectricity has been reported in metal–ferroelectric–ferroelectric*–metal (MFF*M) structures, where switching in the lower‐barrier ferroelectric film locally reduces the switching barrier in the adjacent layer, enabling cooperative switching.^[^ ^7^ ^]^ Applying this mechanism to the AlScN/HfZrO_2_ bilayer suggests that polarization nuclei originating in HfZrO_2_ can trigger polarization switching in adjacent AlScN. However, the extent of switching in AlScN is limited by the magnitude of polarization in HfZrO_2_, resulting in a bilayer *P*_r_ equivalent to that of the HfZrO_2_ single film. Besides, these results suggest that ferroelectric polarization in HfZrO_2_ is effectively screened by the adjacent AlScN film, stabilizing the ferroelectric response without requiring external charge injection, in contrast to the Al_2_O_3_/HfZrO_2_ system.

This work employs the following methods to investigate proximity‐induced switching in AlScN/HfZrO_2_ bilayers.

## 3 Polarization Switching of the AlScN within the AlScN/HfZrO_2_ Bilayer Structure

The chemical reactivity of the AlScN film in KOH solution is known to depend on its polarization direction, exhibiting a significantly higher etch rate when polarized downward.^[^ ^5^, ^12^ ^]^ This polarization‐dependent etching behavior allows the investigation of whether ferroelectric switching in HfZrO_2_ can modulate the polarization direction of AlScN in the AlScN/HfZrO_2_ bilayer.

**Figure 2:**
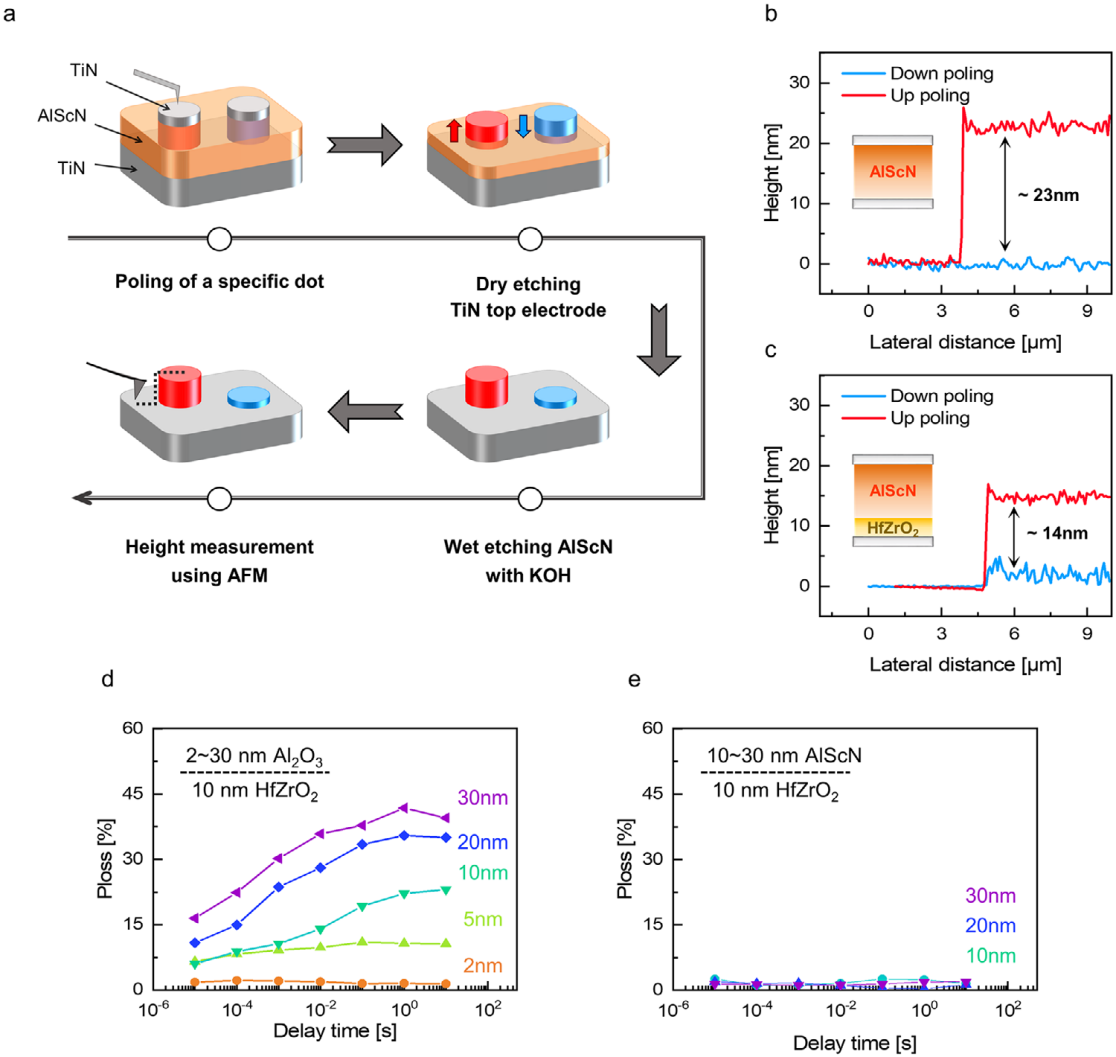
a) Process flow illustrating the evaluation of polarization‐dependent wet etch rates of AlScN film. The film was polarized downward or upward before the etching process. AFM step height profiles of b) 30 nm thick AlScN and c) 30 nm thick AlScN/10 nm‐thick HfZrO_2_ bilayer after wet etching. In both samples, the AlScN film pre‐poled in the downward direction exhibited a greater etch rate than those poled in the upward direction. The proportion of back‐switched domains to total domains in the d) Al_2_O_3_/HfZrO_2_ and e) AlScN/HfZrO_2_ bilayer structures as a function of delay time.

Initially, the polarization‐dependent etch rate of an AlScN film was quantified using a TiN/30 nm‐thick AlScN/TiN capacitor. **Figure** **2**a shows the corresponding wet etching process flow. Specific dot regions were first electrically switched using a 27 V pulse. Subsequently, the TiN top electrode was removed by dry etching, and the exposed AlScN film was etched in KOH solution at 50 °C for 30 s. Surface profiles were then characterized using atomic force microscopy (AFM).

Figure S3a,b of the Supporting Information shows AFM images of the etched AlScN surface. As shown in Figure 2b, the downward‐polarized AlScN exhibited no step height, indicating complete removal by KOH. In contrast, the upward‐polarized film exhibited a ≈23 nm step height, indicating that only ≈7 nm was etched. These results confirm that KOH etching can serve as an effective probe for determining the polarization direction of AlScN.

A similar wet etching experiment was performed on a TiN/30 nm thick AlScN/10 nm‐thick HfZrO_2_/TiN capacitor. A 12 V bias (corresponding to 6 MV cm^−1^) was applied to polarize the AlScN/HfZrO_2_ bilayer, which is insufficient to switch AlScN without the proximity effect. As shown in Figure 2c, the AlScN within the bilayer exhibited noticeable etch rate contrast depending on poling direction, indicating that switching in HfZrO_2_ effectively influenced the polarization state of the adjacent AlScN film. These findings provide direct experimental evidence for proximity‐induced ferroelectricity across the AlScN/HfZrO_2_ interface.

However, compared to the single‐layer AlScN (≈23 nm thickness difference), the bilayer showed a lower thickness difference of ≈14 nm. This reduction suggests that polarization switching in AlScN was partially achieved and constrained by the *P*_r_ of HfZrO_2_. Post‐etch AFM images revealed smooth surfaces for both polarization states, indicating uniform polarization suppression rather than the formation of differently polarized domains (Figure S3c,d of the Supporting Information).

Due to their distinct switching mechanisms, the MDFM and MFF^*^M structures are expected to exhibit different polarization stability. In the MDFM structure, increasing the dielectric layer thickness suppresses charge injection, thereby enhancing depolarization fields and promoting polarization back‐switching.^[^ ^13^ ^]^ Conversely, MFF^*^M comprises two ferroelectric layers whose polarization charges can mutually compensate, mitigating depolarization effects and stabilizing the overall polarization state.

To quantify this difference, polarization loss (*P*_loss_) was evaluated as a function of delay time (*t*_del_) for Al_2_O_3_/HfZrO_2_ and AlScN/HfZrO_2_ capacitors using the pulse sequence shown in Figure S4 of the Supporting Information. Initially, 1 µs length trapezoidal pulses (*P*_S_ and *U*_S_) were applied to determine the amount of switched polarization. Following a variable *t*_del_ (from 1 µs to 10 s), a second pulse set (*P*_BS_ and *U*_BS_) measured the extent of back‐switched domains during *t*_del_. *P*_loss_ was defined as the ratio of the back‐switched domains to the initially switched value.

Figure 2d shows that the *P*_loss_ in the Al_2_O_3_/HfZrO_2_ bilayers increased with *t*_del_, indicating progressive polarization back‐switching driven by depolarization fields.^[^ ^13^, ^14^ ^]^ In contrast, Figure 2e shows that AlScN/HfZrO_2_ bilayers maintain a low *P*_loss_ (≈2%) regardless of *t*_del_ and AlScN thickness. These findings suggest that mutual screening between the ferroelectrics in the MFF^*^M structure effectively suppresses depolarization fields, ensuring superior polarization stability.

## 4 The Unique Ferroelectric Characteristics of AlScN/HfZrO_2_ and AlN/HfZrO_2_ Bilayers

**Figure 3:**
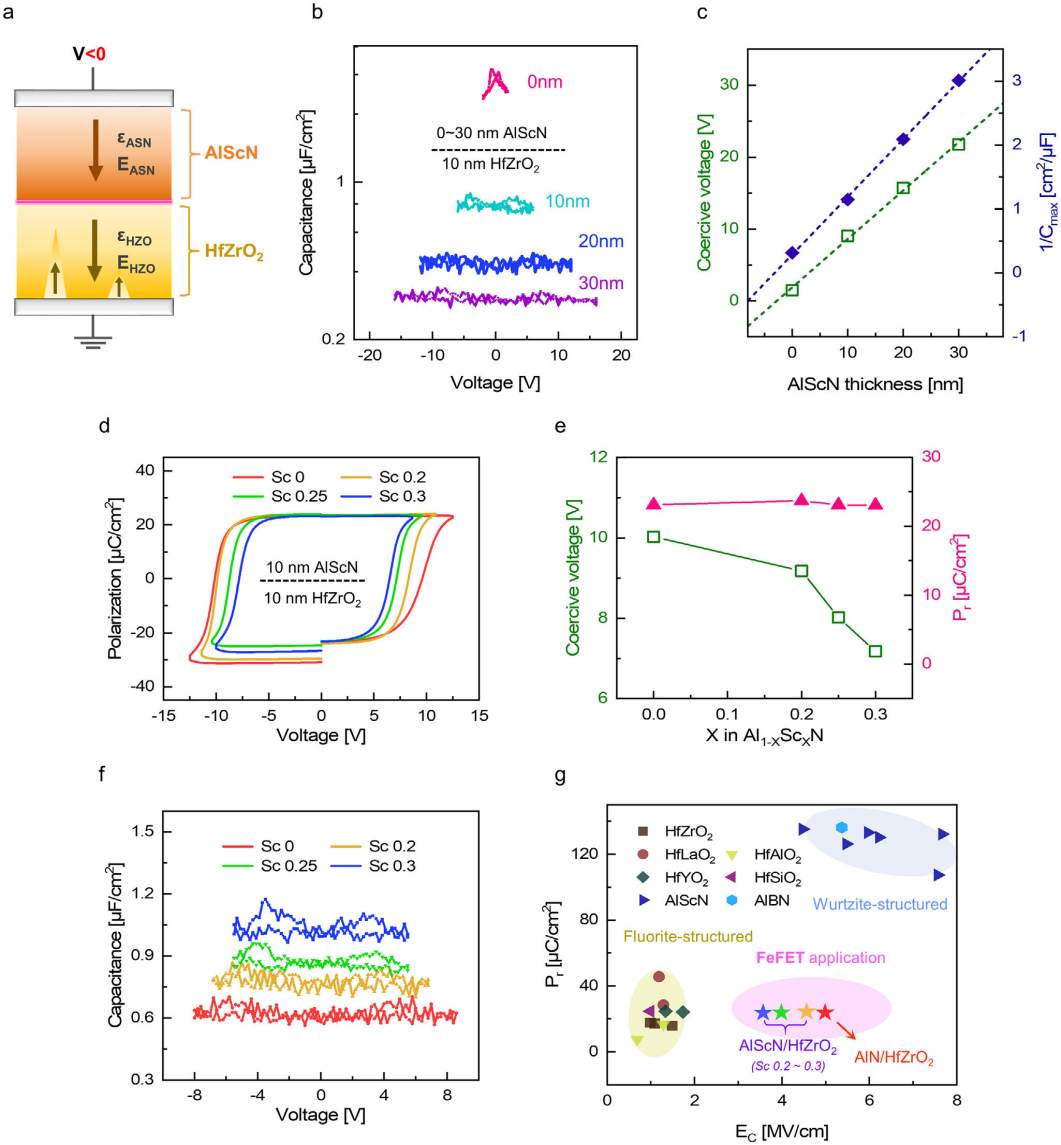
a) Schematic illustration of the AlScN/HfZrO_2_ bilayer at the onset of polarization switching in the HfZrO_2_ film. b) *C*–*V* characteristics of AlScN/HfZrO_2_ bilayers with varying AlScN film thickness. c) Variations in *V*_C_ and 1/*C*_max_ of AlScN/HfZrO_2_ bilayer as a function of AlScN film thickness. d) Evolution of *P–V* curves and e) the corresponding changes in *V*_C_ and *P*_r_ of AlScN/HfZrO_2_ bilayers with *S*c concentrations ranging from 0 to 0.3. f) *C–V* characteristics of AlScN/HfZrO_2_ bilayers with varying *S*c content. g) Comparative analysis of *P*_r_ and *E*_c_ among different ferroelectric materials.^[^ ^5^, ^16^ ^]^ The AlScN/HfZrO_2_ and AlN/HfZrO_2_ bilayer structures exhibit a unique combination of low *P*_r_ and high *E*_C_, distinguishing them from previously reported ferroelectrics.

When proximity ferroelectricity governs switching behavior, the *V*_C_ is determined by the dielectric constants of the constituent ferroelectric layers. As illustrated in **Figure** **3**a, when switching is initiated in HfZrO_2_ of an AlScN/HfZrO_2_ bilayer, *V*_C_ can be expressed as:

(1) $$V_{C}=E_{\mathrm{HZO}}t_{\mathrm{HZO}}+E_{\mathrm{ASN}}t_{\mathrm{ASN}}$$

where *E*_HZO_ (*E*_ASN_) denotes the electric fields across HfZrO_2_ (AlScN) film during switching, and *t*_HZO_ (*t*_ASN_) represents the thickness of HfZrO_2_ (AlScN) film. In the absence of extrinsic charge effects such as charge injection, the electric field distribution follows:

(2) $$\frac{E_{\mathrm{ASN}}}{E_{\mathrm{HZO}}}=\frac{\varepsilon_{\mathrm{HZO}}}{\varepsilon_{\mathrm{ASN}}}$$

where *ε*_HZO_ (*ε*_ASN_) represents the dielectric constant of the HfZrO_2_ (AlScN) film.

The capacitance–voltage (*C*–*V*) measurements were performed on AlScN/HfZrO_2_ bilayers to validate this relationship. As shown in Figure 3b, the *C*–*V* curves exhibit butterfly shapes arising from domain wall motion and nonlinear dielectric response associated with ferroelectric switching. The maximum capacitance (*C*_max_) at the switching point was used to extract the dielectric constant ratio using:

(3) $$\frac{1}{C_{\max}}=\frac{1}{C_{\mathrm{HZO}}}+\frac{1}{C_{\mathrm{ASN}}}=\frac{t_{\mathrm{HZO}}}{\varepsilon_{0}\varepsilon_{\mathrm{HZO}}}+\frac{t_{\mathrm{ASN}}}{\varepsilon_{0}\varepsilon_{\mathrm{ASN}}}$$

Figure 3c shows that 1/*C*_max_ increased linearly with *t*_ASN_, consistent with Equation 3, yielding *ε*_HZO_ ≈ 40 and *ε*_ASN_ ≈ 12. *V*_C_ also increased linearly with *t*_ASN_, following Equation 1. The extracted electric fields were *E*_HZO_ ≈ 1.9 MV cm^−1^ and *E*_ASN_ ≈ 6.7 MV cm^−1^, resulting in an electric field ratio of *E*_ASN_/*E*_HZO_ ≈ 3.5, which closely matches the dielectric constant ratio of *ε*_HZO/_ *ε*_ASN_ ≈ 3.3. These results confirm that the electric field distribution across each layer in the MFF*M structure is primarily governed by its respective dielectric constant.

Based on this understanding, the *S*c concentration in the AlScN film was varied to modulate the *V*_C_ of the AlScN/HfZrO_2_ bilayer. Since *ε*_ASN_ increases with Sc content, the contrast between *ε*_HZO_ and *ε*_ASN_ is expected to decrease.^[^ ^15^ ^]^ Consequently, an increase in Sc content should lead to a reduction in the *E*_ASN_, decreasing the *V*_C_ of the bilayer.

Figure 3d shows the *P–V* hysteresis loops of 10 nm thick AlScN/10 nm thick HfZrO_2_ bilayers with Sc concentrations in the AlScN film ranging from 0 to 0.3. Robust ferroelectric switching occurred across all compositions, with *P*_r_ remaining constant at the HfZrO_2_ value, as shown in Figure 3e. These results indicate that the proximity ferroelectricity mechanism, driven by HfZrO_2_ switching, was preserved regardless of the *S*c content. Notably, even in a pure AlN film, where the switching barrier has been reported to exceed the dielectric breakdown field, ferroelectric switching became feasible within the bilayer, highlighting the effectiveness of proximity‐induced switching.^[^ ^8a^ ^]^

Figure 3f shows the *C*–*V* curves of AlScN/HfZrO_2_ bilayers with varying *S*c concentrations. The capacitance value increased with *S*c content due to the increased *ε*_ASN_. Consequently, *E*_ASN_ decreased with increasing *S*c content, leading to a gradual reduction in *V*_C_ of the bilayer, as shown in Figure 3e.

Figure 3g compares *P*_r_ and *E*_C_ across various ferroelectric materials.^[^ ^5^, ^16^ ^]^ The AlScN/HfZrO_2_ bilayer uniquely maintains a low *P*_r_ comparable to fluorite systems while achieving high *E*_C_. As the *S*c concentration decreased, the *E*_C_ of the bilayer increased accordingly, reaching a maximum of ≈5 MV cm^−1^ in the AlN/HfZrO_2_ structure. These unique ferroelectric characteristics of the bilayer are particularly advantageous for FeFETs, where conventional ferroelectrics face severe challenges. For instance, the low *E*_C_ of fluorite‐structured materials restricts the attainable memory window.^[^ ^3^ ^]^ In contrast, wurtzite‐structured ferroelectrics exhibit high *E*_C_ exceeding 6 MV cm^−1^, theoretically enabling wider memory windows. Nevertheless, their exceptionally high *P*_r_ could induce excessive charge injection and interfacial trapping during switching, compromising memory stability. Therefore, this bilayer structure exhibits promising ferroelectric properties, making it suitable for the realization of high‐performance FeFETs.

## 5 Impact of Ferroelectric Gate Stack on the Memory Window of FeFETs

As discussed earlier, the excessively high *P*_r_ of AlScN could cause excessive charge injection during FeFET operation, which in turn degrades memory stability. To investigate these limitations, an n‐type FeFET was fabricated using a 30 nm thick AlScN/0.7 nm thick SiO_2_ ferroelectric gate stack (ASN FeFET). Figure S5 in the Supporting Information illustrates the detailed fabrication process with a gate length and width of 5 µm. In FeFET, the complete suppression of the SiO_2_ layer between the ferroelectric oxide layer and the Si channel, which degrades the device's reliability, is challenging.^[^ ^17^ ^]^ Therefore, to prevent excessive SiO_2_ growth during ferroelectric deposition and annealing, a 0.7 nm thick SiO_2_ film was chemically grown before depositing the ferroelectric gate stack. The *P*–*V* hysteresis curve measured by connecting the substrate and source/drain terminals confirmed strong switching with P_r_ exceeding 100 µC cm^−2^ (Figure S6a of the Supporting Information). However, the DC double‐sweep transfer characteristics showed a clockwise hysteresis loop, indicative of trap‐dominated behavior (Figure S6b of the Supporting Information). This behavior is attributed to the large *P*_r_ of AlScN, which induced a strong electric field across the SiO_2_ film during switching. This electric field facilitated excessive charge injection through SiO_2_, resulting in trap charges that exceeded the ferroelectric polarization.^[^ ^10a^ ^]^ As a result, the memory window anticipated from ferroelectric charge was canceled out, resulting in the clockwise hysteresis. These results highlight a critical limitation of employing AlScN film as a ferroelectric layer in FeFET devices.

In contrast, the AlScN/HfZrO_2_ bilayer mitigates this issue by combining high *V*_C_ with low *P*_r_. Specifically, when the *S*c concentration was decreased to zero (pure AlN), the bilayer showed the highest *V*_C_ value. Consequently, the FeFET showed the largest memory window when the AlN/HfZrO_2_ bilayer was used as the ferroelectric gate stack, as shown in Figure S7 of the Supporting Information. Based on these findings, the following discussion focuses on FeFETs employing three different ferroelectric gate stacks.

- 10 nm thick HfZrO_2_/0.7 nm thick SiO_2_: HZO FeFET,
- 10 nm thick Al_2_O_3_/10 nm thick HfZrO_2_/0.7 nm thick SiO_2_: AO/HZO FeFET,
- 10 nm thick AlN/10 nm thick HfZrO_2_/0.7 nm thick SiO_2_: AlN/HZO FeFET.

**Figure 4:**
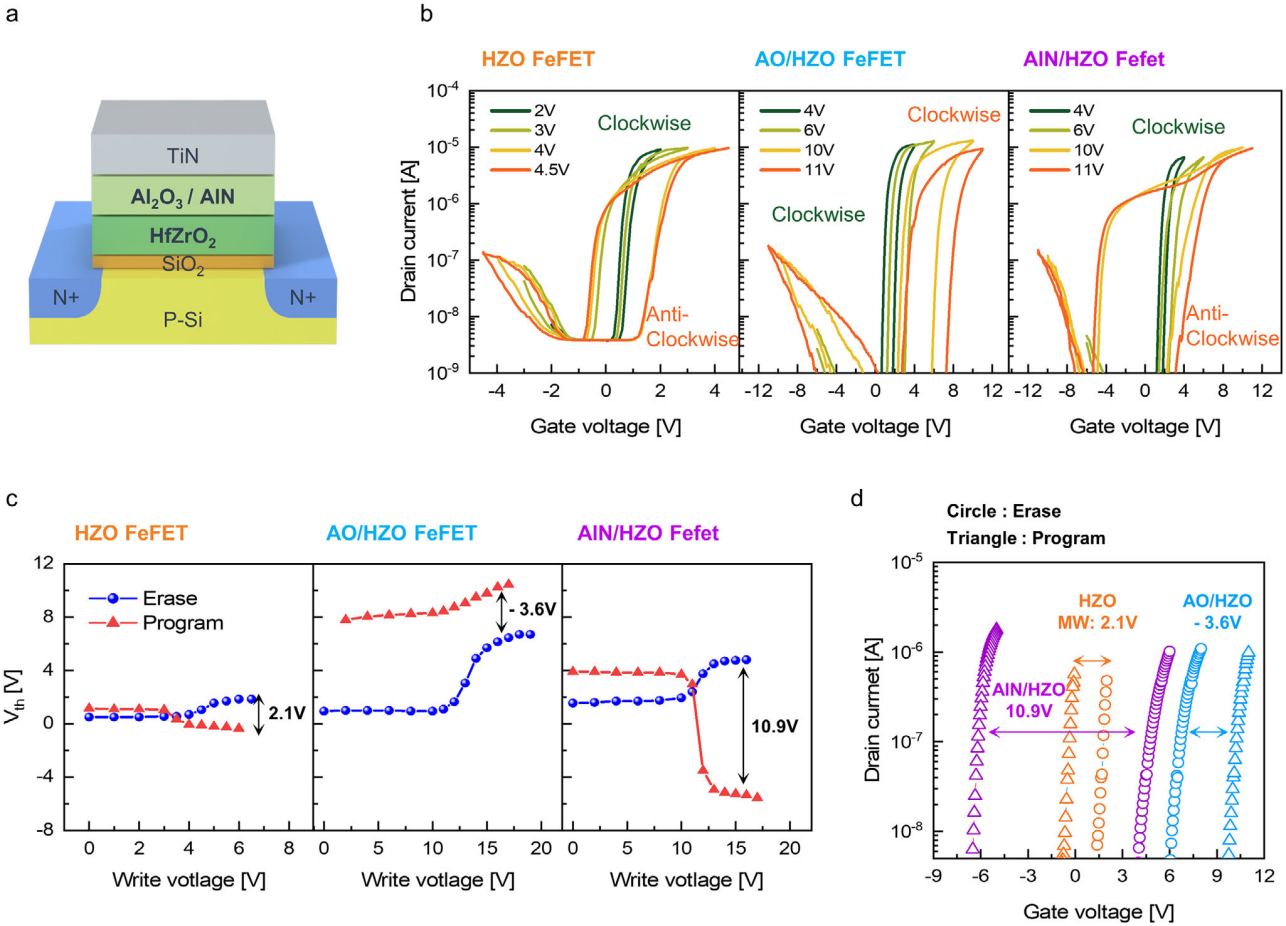
a) Schematic illustration of FeFET devices. b) *I*_D_–*V*_G_ hysteresis curves of HZO FeFET, AO/HZO FeFET, and AlN/HZO FeFET devices. c) *V*_th_ variations of FeFETs with different ferroelectric gate stacks as a function of erase and program pulse voltages (pulse width: 100 µs). d) *I*_D_–*V*_G_ characteristics in the erase and program states for FeFETs with different ferroelectric gate stacks.

**Figure** **4**a shows the schematic illustration of the FeFET devices. Figure S8 of the Supporting Information shows the output characteristics of the device with linear and saturation regions. Figure 4b shows the DC double‐sweep transfer curves of the devices, and both HZO FeFET and AlN/HZO FeFET exhibited anti‐clockwise hysteresis loops, indicative of ferroelectric switching. In contrast, the AO/HZO FeFET showed a clockwise hysteresis loop, even under high sweep voltages. This result confirms a charge‐trap‐dominated behavior of the AO/HZO FeFET, similar to the ASN FeFET.

Subsequent FeFET performance was examined using the industry‐standard incremental step pulse programming (ISPP) method. 100 µs length negative pulses were applied for erase, followed by positive program pulses. The threshold voltage (*V*_th_) was extracted using the constant current method at the drain current of 10^−7^ A. Figure 4c shows the *V*_th_ shifts as a function of erase and program voltages. In FeFET, ferroelectric switching leads to an increase (decrease) in the *V*_th_ following the erase (program) operation.^[^ ^3a^ ^]^

The HZO FeFET showed a ferroelectric polarization‐induced memory window. However, due to the low *V*_C_ of the HfZrO_2_, the memory window was limited to ≈2.1 V. In contrast, the AO/HZO FeFET showed a large initial *V*_th_ increase (≈6 V) after erase, consistent with the direction expected from ferroelectric switching. This significant shift could be attributed to both the high *V*_C_ of the Al_2_O_3_/HfZrO_2_ bilayer and the electrons trapped at the Al_2_O_3_/HfZrO_2_ interface. However, during the subsequent program, *V*_th_ increased unexpectedly, indicating trap‐dominated behavior. Previous studies have reported that holes trapped at the Al_2_O_3_/HfZrO_2_ interface could rapidly detrap during the program, increasing *V*_th_.^[^ ^18^ ^]^ As a result, the AO/HZO FeFET exhibited a ≈3.6 V memory window driven by charge trapping effect (clockwise hysteresis curve).

In contrast, the AlN/HZO FeFET exhibited a wide and stable memory window of ≈10.9 V, attributed to the high *V*_C_ and the absence of trap charges between the two layers. Figure 4d shows the device's drain current–gate voltage (*I*_D_–*V*_G_) characteristics in erase and program states. In addition, Figure S9 of the Supporting Information further confirms that the AlN/HZO FeFET maintains a memory window exceeding 10 V even under 1 µs length short pulses, validating its potential for high‐speed operation.

## 6 Superior Reliability of AlN/HZO FeFET

**Figure 5:**
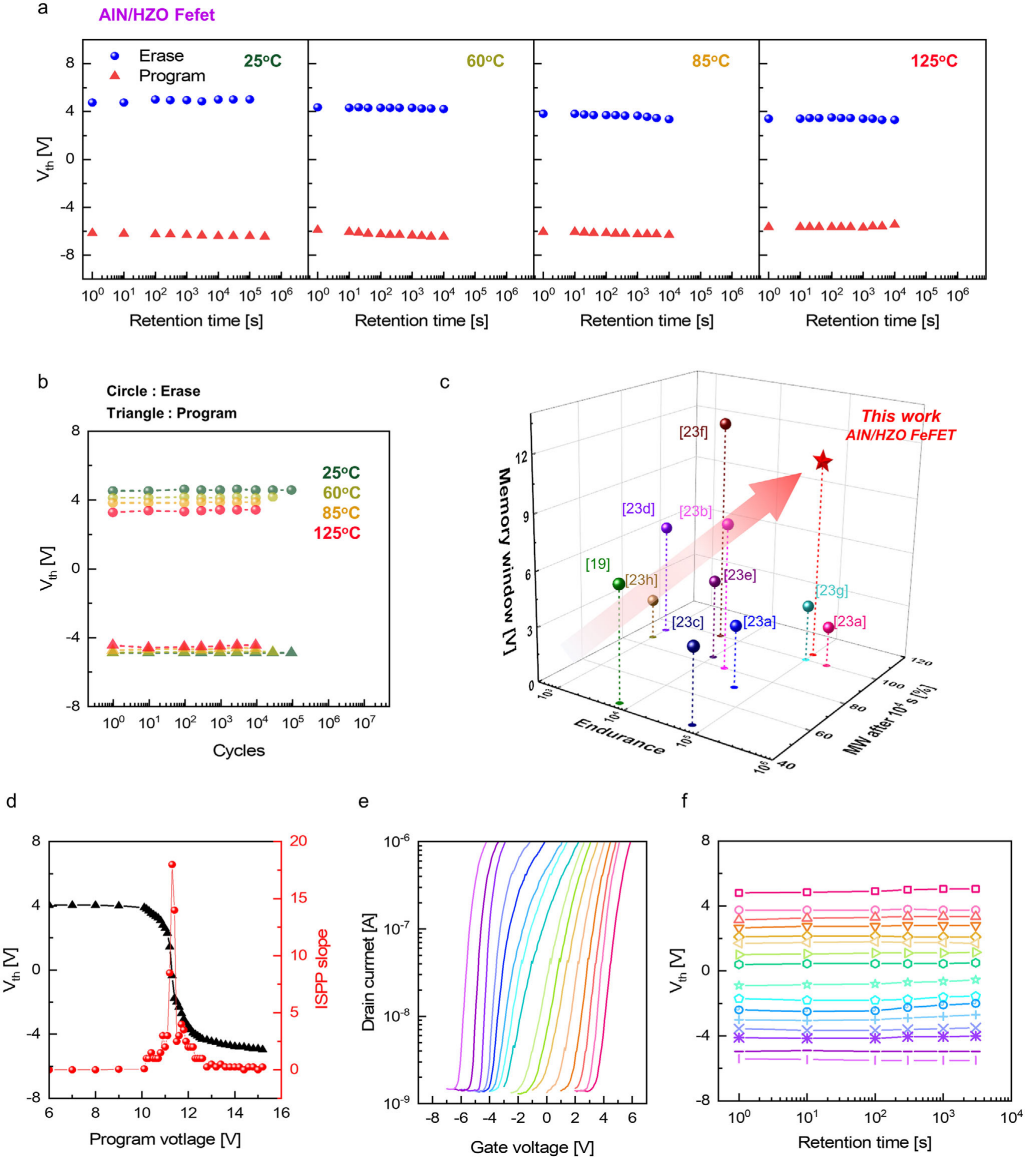
a) Retention characteristics of AlN/HZO FeFET measured over an extended time. b) Endurance performance of AlN/HZO FeFET. The retention and endurance tests were conducted while varying the temperature from 25 to 125 °C. The device operates up to 10^5^ cycles stably at room temperature, which satisfies the current NAND‐type storage applications. Operating the devices with cycles exceeding 10^5^ induced a hard breakdown. c) Benchmarking of memory window, endurance, and retention performance of AlN/HZO FeFET against reported FeFETs.^[^ ^19^, ^23^ ^]^ d) *V*_th_ evolution of the AlN/HZO FeFET during ISPP measurement. e) *I*_D_–*V*_G_ characteristics of 16 distinguishable states and f) their corresponding *V*_th_ stability over time.

Retention and endurance tests were conducted to evaluate the reliability of FeFETs. Figure S10a,b of the Supporting Information show the pulse sequences used for retention and endurance measurements, respectively. Retention characteristics of HZO FeFET and AO/HZO FeFET are shown in Figure S10c,d of the Supporting Information. The AO/HZO FeFET exhibited a significant V_th_ shift over time, attributed to the instability of trapped charges at the Al_2_O_3_/HfZrO_2_ interface.^[^ ^18^, ^19^ ^]^ In contrast, the AlN/HZO FeFET maintained a stable memory state over extended durations attributed to the high *V*_C_ of the AlN/HfZrO_2_ bilayer, as shown in **Figure** **5**a. Retention measurements at elevated temperatures up to 125 °C further demonstrated thermal robustness. While a slight reduction in memory window was observed with increasing temperature due to the temperature dependence of *V*_C_, the AlN/HZO FeFET retained stable performance under these high‐temperature conditions.^[^ ^20^ ^]^

Figure S10e,f of the Supporting Information compare the endurance characteristics of HZO FeFET and AO/HZO FeFET, respectively. While the HZO FeFET operated reliably up to 10^6^ cycles, the AO/HZO FeFET failed after only 10 cycles due to accumulated damage in the Al_2_O_3_ film from repeated charge injection.^[^ ^18^, ^19^ ^]^ In contrast, the AlN/HZO FeFET maintained stable switching over 10^5^ cycles without memory window degradation and maintained robust performance up to 10^4^ cycles at 125 °C, as shown in Figure 5b. Figure S11a,b of the Supporting Information show the *P*–*V* curve and endurance characteristics of the TiN/10 nm thick AlN/10 nm‐thick HfZrO_2_/TiN capacitor, indicating that *P*_r_ decreased by ≈30% after 2×10^6^ cycles. A recent study has revealed that nitrogen vacancies at the AlSN interface not only degrade its dielectric properties but also increase the switching barrier, leading to non‐switchable dead layer formation.^[^ ^21^ ^]^ Therefore, for the AlN/HfZrO_2_ bilayer, an increase in nitrogen vacancies at the AlN interface during field cycling could cause the fatigue phenomenon and premature breakdown. A detailed analysis of this fatigue behavior will be further investigated in future studies. Figure S11c of the Supporting Information shows that the AlN/HZO FeFET device has lower endurance compared to the capacitor structure due to the presence of the SiO_2_ layer between HfZrO_2_ and the Si channel. This reliability issue caused by the SiO_2_ layer can be improved by inserting a high‐*k* dielectric layer between the ferroelectric layer and the Si channel or by using highly active metal gates to leverage oxygen scavenging effects and minimize SiO_2_ formation.^[^ ^22^ ^]^

Figure 5c benchmarks the AlN/HZO FeFET against previously reported Si‐channel FeFETs regarding memory window, endurance, and retention.^[^ ^19^, ^23^ ^]^ Retention was evaluated based on the percentage of memory window maintained after 10^4^ s. The AlN/HZO FeFET achieved a wide memory window while simultaneously achieving excellent endurance and retention characteristics.

Finally, the ISPP method was employed to evaluate the multi‐level cell capability of the AlN/HZO FeFET. Figure 5d shows the *V*_th_ shifts as a function of program voltage, revealing a maximum ISPP slope of 18 near 11.3 V program voltage. Figure 5e,f show 16 distinct states generated via ISPP and their retention characteristics. All states remained well separated and stable over time, confirming the suitability of AlN/HZO FeFET for reliable quad‐level cell operation.

## 7 Conclusion

This study investigated the polarization‐switching behavior in a bilayer structure composed of AlScN (or AlN) and HfZrO_2_ films. Wet etching and electrical measurements demonstrated that polarization switching initiated in HfZrO_2_ induced polarization reversal in the adjacent AlScN film. This cooperative switching enabled the AlScN/HfZrO_2_ bilayer to exhibit a unique combination of low *P*_r_ and high *E*_C_, which is not attainable in any single‐phase ferroelectric material.

A FeFET incorporating a 10 nm thick AlN/10 nm thick HfZrO_2_ ferroelectric gate stack was fabricated to leverage these properties. The device exhibited a wide memory window exceeding 10 V and outstanding retention and endurance characteristics. The memory performance remained robust even at elevated temperatures up to 125 °C. These findings highlight the potential of integrating structurally dissimilar ferroelectrics to engineer polarization behavior, thereby opening new avenues for developing ferroelectrics with designer properties tailored for advanced device applications.

## 8 Experimental Section

### Capacitor Fabrication

50 nm thick TiN bottom electrodes were deposited on SiO_2_/Si substrates via direct current (DC) sputtering (ENDURA 5500, Applied Materials). Subsequently, HfZrO_2_ thin films were deposited on the substrates by thermal atomic layer deposition (Atomic‐Classic, CN‐1) at a substrate temperature of 285 °C. Hf[N(C_2_H_5_)CH_3_]_4_ (TEMA‐Hf), Zr[N(C_2_H_5_)CH_3_]_4_ (TEMA‐Zr), and ozone were used as the Hf, Zr, and oxygen sources, respectively.

The Al_2_O_3_ films were deposited by radio frequency (RF) magnetron sputtering using an Al_2_O_3_ ceramic target at room temperature, with a power of 400 W and an Ar gas flow of 60 sccm. The working pressure was set to 5 mTorr. The AlScN films were deposited by RF reactive magnetron co‐sputtering using AlN and Sc targets. The deposition was performed at room temperature under a nitrogen atmosphere with a gas flow rate of 20 sccm and a working pressure of 20 mTorr. The sputtering powers for the AlN and Sc targets were set to 290 W and 210 W, respectively, to achieve a Sc concentration of 0.2 in the AlScN films. The Sc content in the AlScN film was adjusted by varying the relative power applied to each target while maintaining a total power of 500 W.

Following the ferroelectric or dielectric film deposition, 50 nm thick TiN top electrodes were deposited by DC reactive sputtering. Post‐metallization annealing was conducted at 500 °C for 30 s to crystallize the HfZrO_2_ film. Circular top electrodes with an area of 1000 µm^2^ were patterned using photolithography (DL‐1000 HP, NanoSystem Solutions) and defined by dry etching (Plasma Pro System 100 Cobra, Oxford Instruments).

### FeFET Fabrication

N‐type FeFETs were fabricated on p‐type Si wafers using a gate‐last CMOS process. The source and drain regions were defined by phosphorus ion implantation, followed by dopant activation through rapid thermal annealing at 1000 °C for 10 s. To prevent excessive growth of the SiO_2_ layer, a 0.7 nm thick interfacial SiO_2_ layer was chemically grown before the deposition of the ferroelectric gate stack. The thickness of the SiO_2_ film was measured by spectroscopic ellipsometry (M‐2000, J.A. Woollam).

The ferroelectric gate stacks were deposited using the same procedure for capacitor fabrication. A 50 nm thick TiN gate electrode was deposited by DC sputtering, followed by annealing at 500 °C for 30 s to crystallize the HfZrO_2_ film into the ferroelectric phase. Gate electrodes were patterned via photolithography and dry etching. Contact holes were then opened in the source and drain regions through dry etching, and a 30 nm‐thick Al contact layer was deposited and patterned to complete the device fabrication.

### Characterization

The thickness of the AlScN films was measured by spectroscopic ellipsometry. The film's crystallographic structure and phase quality were characterized using XRD (X'Pert Pro, PANalytical) and HRTEM (JEM‐2100F, JEOL Ltd). After wet etching, the surface profile of AlScN films was analyzed using AFM (NX10, Park Systems). Electrical measurements, including ferroelectric switching behavior and transfer characteristics of FeFET devices, were performed using a semiconductor parameter analyzer (Keithley 4200A‐SCS).

## Conflict of Interest

The authors declare no conflict of interest.

## Supporting information



Supporting Information

## Data Availability

The data that support the findings of this study are available from the corresponding author upon reasonable request.
