# Supplementary material for: Proximity‐Induced Ferroelectric Switching in Wurtzite/Fluorite Bilayers for High‐Performance Ferroelectric Field‐Effect Transistor
Source: Adv Mater. 2025 Aug 21;37(45):e09088. doi: 10.1002/adma.202509088 (PMC12617061; doi:10.1002/adma.202509088)
Supplement: Supplementary file 1 — Supporting Information [file ADMA-37-e09088-s001.docx]

**Supporting Information**

**Proximity-Induced Ferroelectric Switching in Wurtzite/Fluorite Bilayers for High-Performance Ferroelectric Field-Effect Transistor**

*Kyung Do Kim^1^, Min Kyu Yeom^1^, Han Sol Park^1^, Gwangsik Jeon^1^ and Cheol Seong Hwang^1,a^*

^1^Department of Materials Science and Engineering and Inter-University Semiconductor Research Center, Seoul National University, Seoul 08826, South Korea

^a^ Corresponding author: cheolsh@snu.ac.kr

***Keywords:*** *Ferroelectric, Aluminum scandium nitride, Hafnium zirconium oxide, Proximity ferroelectricity, Heterostructure, Ferroelectric field-effect transistors*

**Supporting Figures**

**
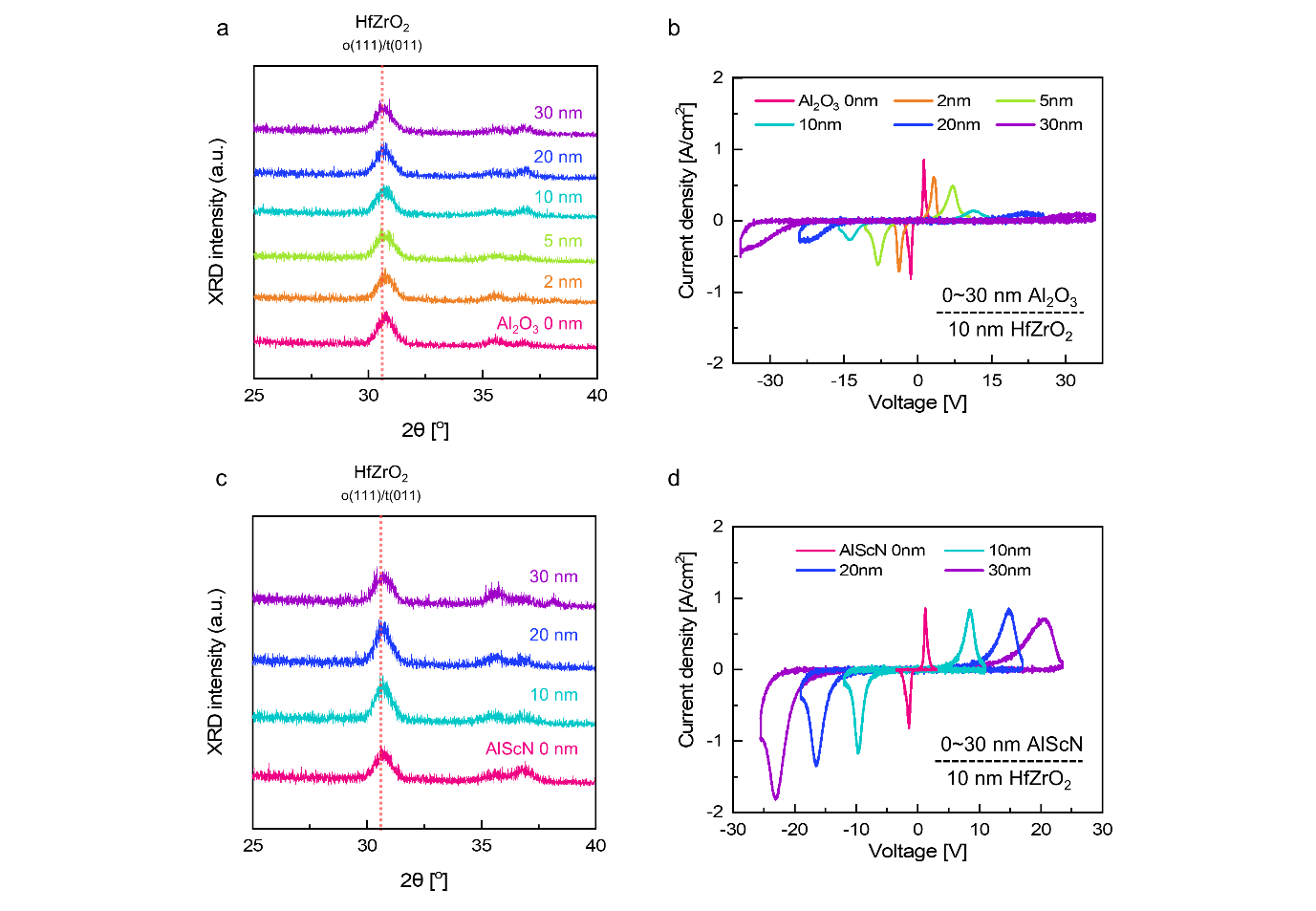
**

**Figure S1.** (a) GIXRD patterns and (b) J-V curves of Al_2_O_3_/HfZrO_2_ bilayers with varying Al_2_O_3_ film thickness. (c) GIXRD patterns and (d) J-V curves of AlScN/HfZrO_2_ bilayers with varying AlScN film thickness.


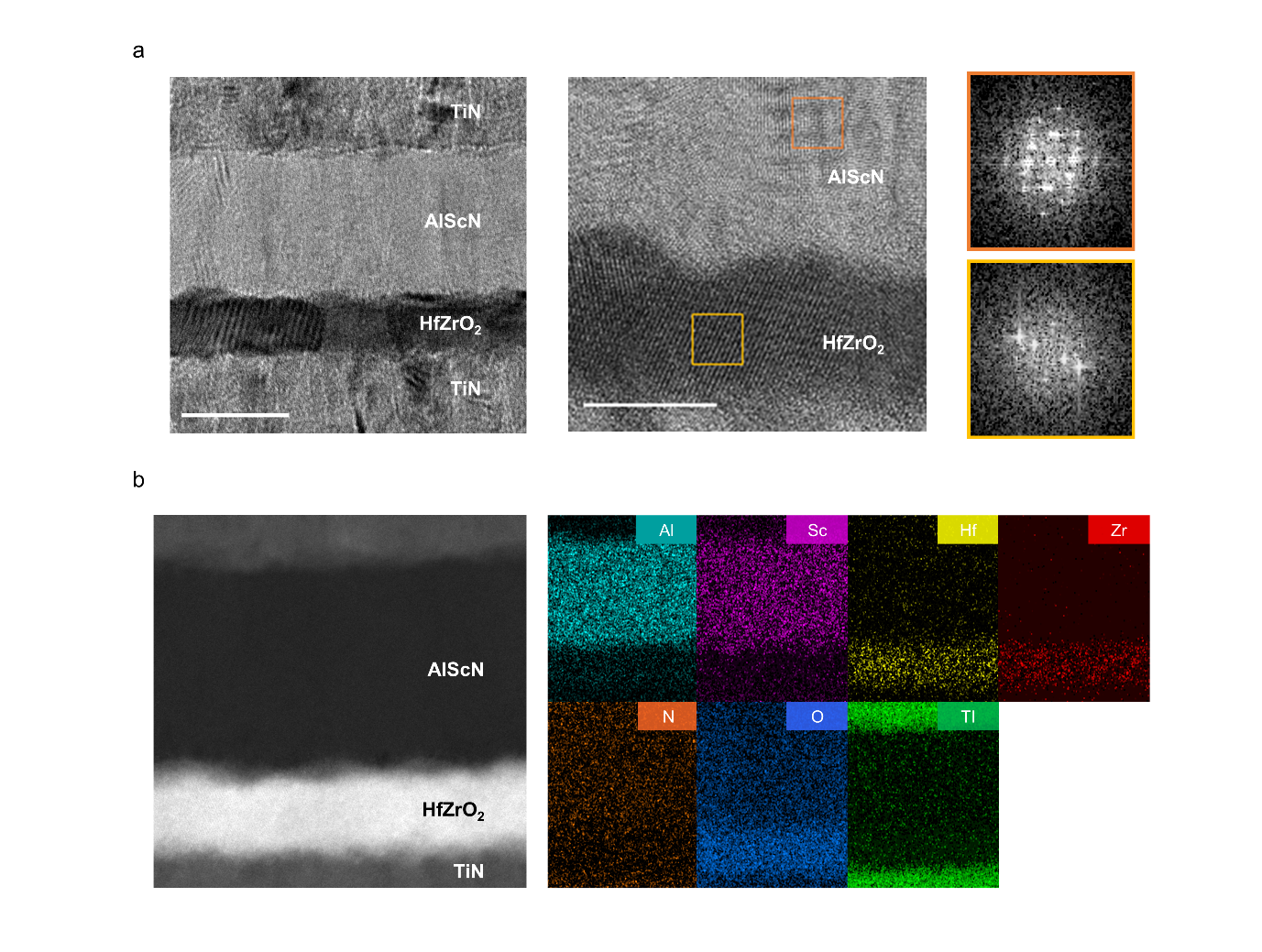


**Figure S2.** (a) Cross-sectional HRTEM image of the 30 nm-thick AlScN/10 nm-thick HfZrO_2_ bilayer structure. Distinct lattice fringes and FFT patterns of the marked regions indicate the crystalline nature of each layer. (b) Cross-sectional EDS elemental mapping obtained from TEM confirms clear separation between the HfZrO_2_ and AlScN layers, with no observable interdiffusion at the interface.


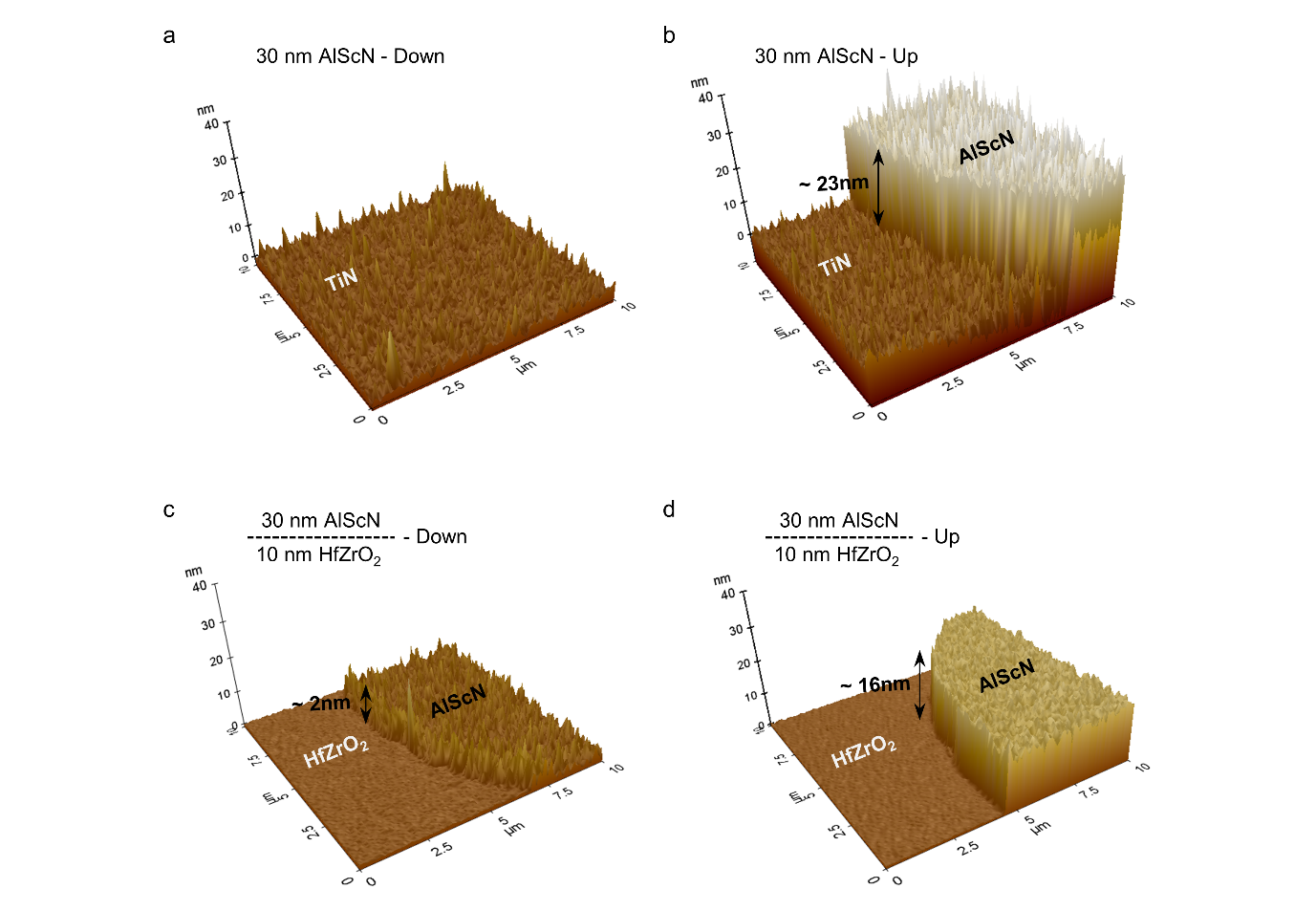


**Figure S3.** (a), (b) AFM images of 30 nm-thick AlScN single-layer films after wet etching. Before etching, the films were electrically poled in the (a) downward and (b) upward directions. (c), (d) AFM images of 30 nm-thick AlScN/10 nm-thick HfZrO_2_ bilayers after wet etching. Before etching, the films were electrically poled in the (c) downward and (d) upward directions.


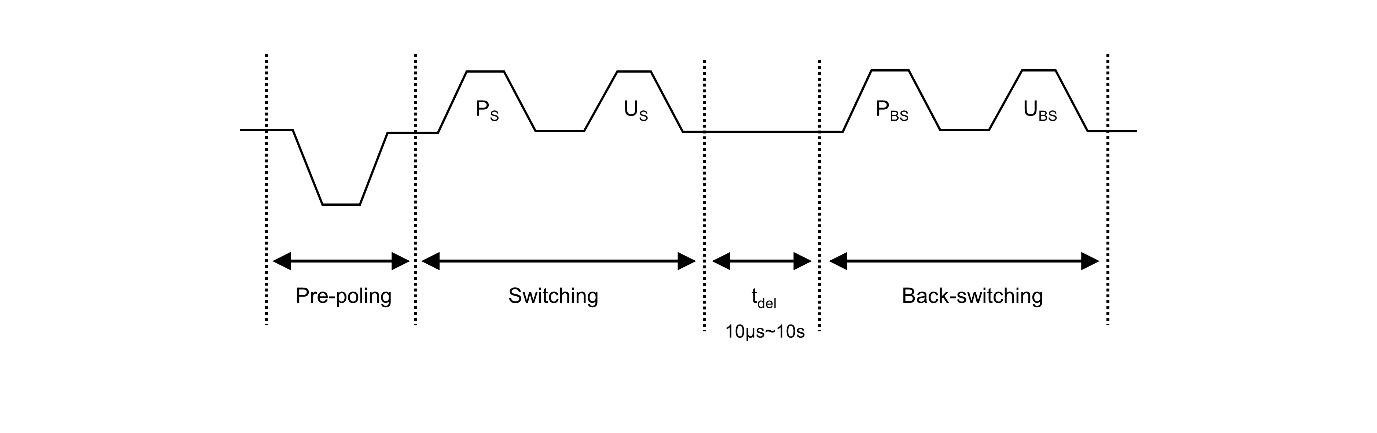


**Figure S4.** Schematic diagram of the pulse sequence used to evaluate the stability of polarization domains.


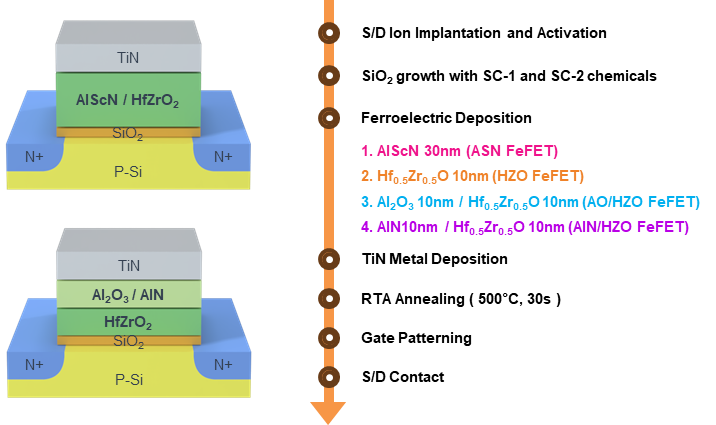


**Figure S5.** Process flow for the fabrication of FeFETs with different ferroelectric gate stacks.


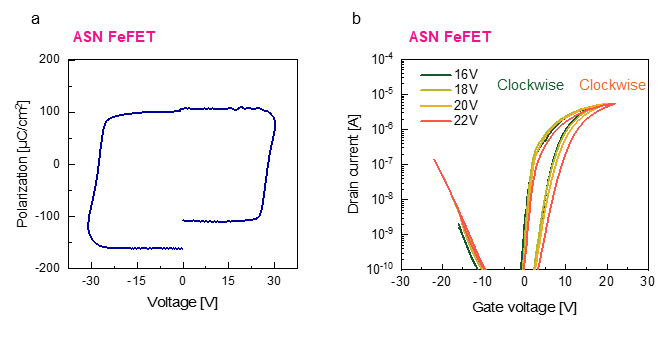


**Figure S6.** (a) P-V curve and (b) I_D_-V_G_ hysteresis curve of ASN FeFET.


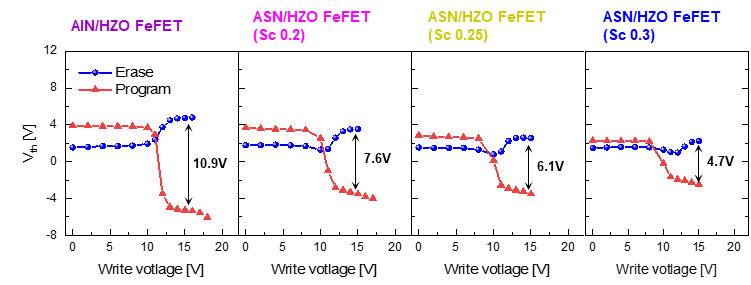


**Figure S7.** V_th_ variations of FeFETs with AlScN/HfZrO_2_ ferroelectric gate stacks as a function of erase and program pulse voltages (pulse width: 100 μs). The Sc concentration was controlled from 0 to 0.3.


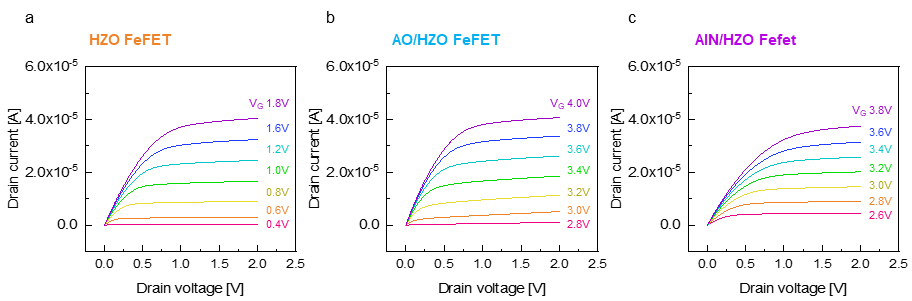


**Figure S8.** Output characteristics curves of (a) HZO FeFET, (b) AO/HZO FeFET, and (c) AlN/HZO FeFET.


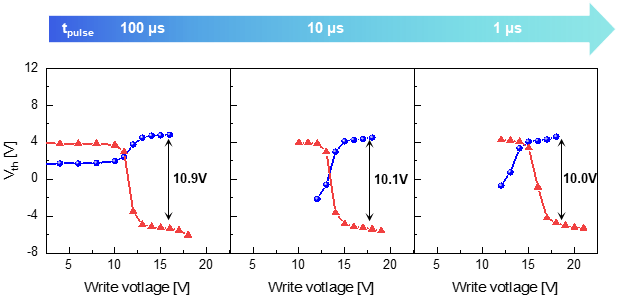


**Figure S9.** Variation in V_th_ of the AlN/HZO FeFET as a function of erase and program pulse voltage, with pulse widths ranging from 1 µs to 100 µs.


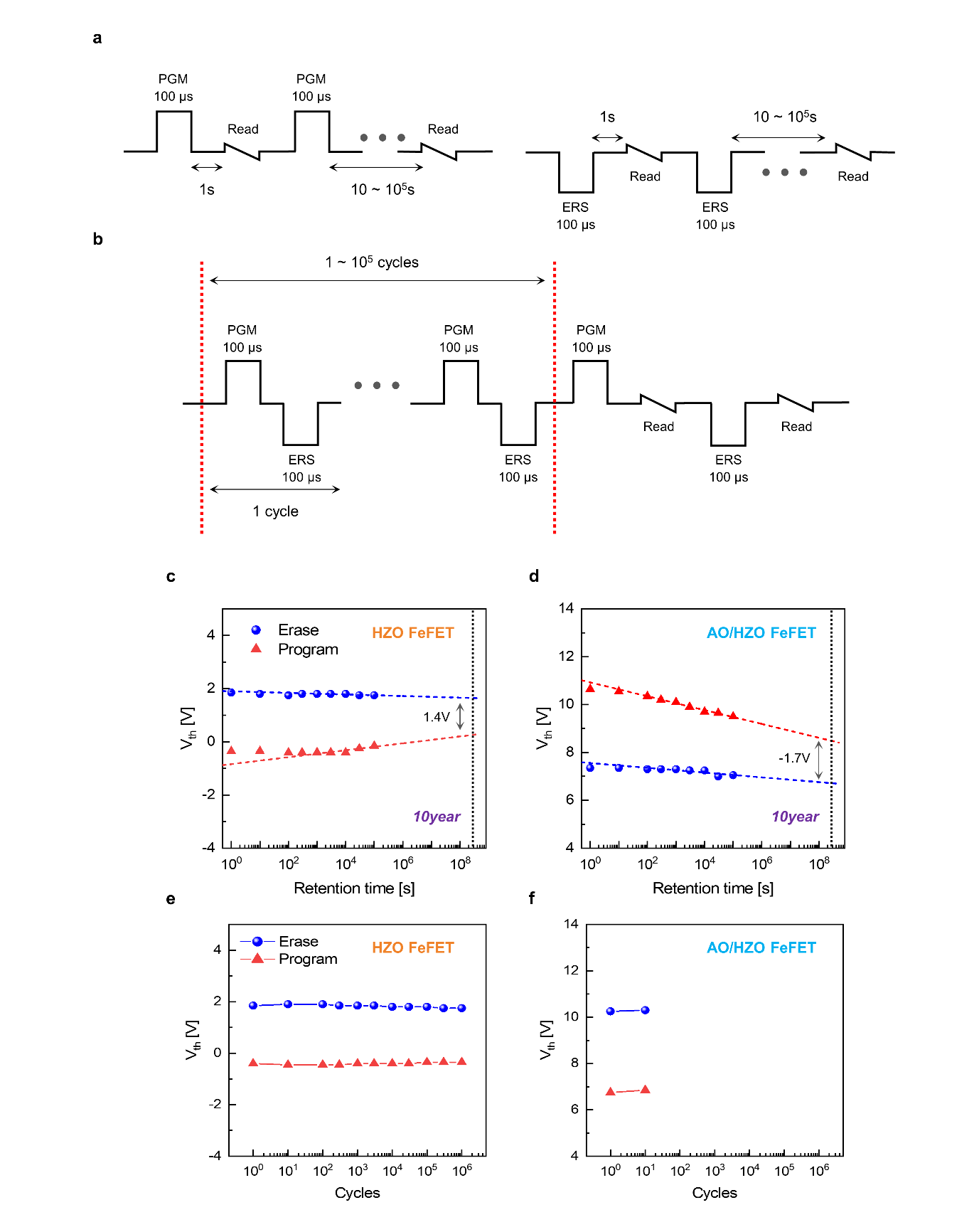


**Figure S10.** Pulse schemes for (a) retention and (b) endurance measurements. Retention characteristics of (c) HZO FeFET and (d) AO/HZO FeFET measured over extended time. Endurance performance of (e) HZO FeFET and (f) AO/HZO FeFET.


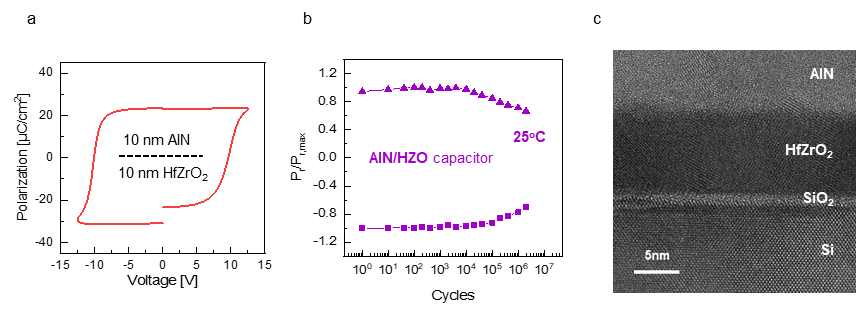


**Figure S11.** (a) P-V curve and (b) endurance characteristics of the TiN/AlN/HfZrO_2_/TiN capacitor. (c) Cross-sectional HRTEM image of the AlN/HZO FeFET.
